# A low level of health literacy is a predictor of corticophobia in atopic dermatitis^⋆^

**DOI:** 10.1016/j.abd.2021.11.007

**Published:** 2022-08-31

**Authors:** Tiago Fernandes Gomes, Katarina Kieselova, Victoria Guiote, Martinha Henrique, Felicidade Santiago

**Affiliations:** Department of Dermatology, Centro Hospitalar de Leiria, Leiria, Portugal

**Keywords:** Dermatitis, Atopic, Health literacy, Steroids, Therapeutics

## Abstract

**Background:**

Topical corticosteroids (TCS) are the mainstay of treatment in atopic dermatitis (AD) flares. The fears and worries concerning TCS are known as corticophobia. Corticophobia is common in patients with AD and can lead to suboptimal TCS application and treatment failure. Health literacy (HL) may influence corticophobia. TOPICOP© and HLS-EU-PT questionnaires have been developed to evaluate corticophobia and HL, respectively.

**Objective:**

Evaluate the relationship between corticophobia and the degree of HL in patients with AD.

**Methods:**

Prospective cross-sectional study with AD patients followed at a Dermatology Department, between September 2019 and February 2020. Patients, or their parents (if patients had ≤ 15 years), were invited to answer TOPICOP© questionnaire, HLS-EU-PT questionnaire, and a disease characterization and demographic questionnaire.

**Results:**

We included 61 patients (57.4% females, mean age 20 ± 13.8 years, mean disease duration of 12.5 ± 11.4 years). TOPICOP© mean score was 44.8 ± 20.0 (8.3 to 88.9) and HLS-EU-PT mean score was 30.5 ± 8.5 (1.1 to 47.9). TOPICOP© score was negatively correlated with HLS-EU-PT score (p = 0.002, *r* = -0.382,  *r^2^* = 0.146). There was no statistical difference between TOPICOP© score and disease characteristics (disease severity, family history of AD or personal history of other atopic diseases).

**Study limitations:**

Small and heterogenous cohort composed of patients and patients’ parents.

**Conclusion:**

The degree of corticophobia is similar to the values reported in other studies. HL had an inverse correlation with corticophobia. Lower HL was shown to be a predictor of higher corticophobia. The promotion of health literacy is essential for the correct use of TCS and good control of AD.

## Introduction

Atopic dermatitis (AD) is a chronic relapsing inflammatory skin disorder with a significant impact on the quality of life.^1^ Globally, its prevalence is increasing, affecting up to 20% of children and 10% of adults.^2^, ^3^, ^4^, ^5^ Topical anti-inflammatory therapies are the first step of treatment in AD flares.^6^, ^7^ Topical corticosteroids (TCS) are the mainstay of treatment in most patients with AD and they often require long-term treatment with TCS for many years. Poor adherence to treatment is common in AD and can lead to treatment failure.^8^, ^9^, ^10^ TCS resistance may be partially due to non-adherence because of TCS phobia.^11^ TCS phobia, also known as corticophobia, defines the negative feelings and beliefs related to TCS experienced by patients and their caregivers. The prevalence of corticophobia in AD ranges from 21% to 84%.^12^ Health literacy is the term used to describe people's capacities to meet the complex demands related to health in modern society. It results from health education and communication activities and determines the individual ability to gain access to, understand, and use information in order to promote and maintain good health.^13^, ^14^ The present group of authors proposed to study the effect of health literacy on corticophobia.

## Methods

### Patients and setting

We conducted a prospective cross-sectional study at the Department of Dermatology of a level 2 Hospital in Portugal, between September 2019 and February 2020. Patients with atopic dermatitis, with at least 2 Dermatology appointments were included. We defined two groups according to the age of the patient: ≤ 15 years and > 15 years.

### Questionnaires

Parents of the patients with ≤ 15 years and the patients with >15 years were invited to answer three questionnaires: TOPICOP© questionnaire, HLS-EU-PT questionnaire, and a disease characterization and demographic questionnaire.

TOPICOP© is a validated scale for the assessment of TCS phobia among AD patients and their parents.^15^ The feasibility and comprehensibility of the TOPICOP© score have been addressed across different countries.^16^ The original version is in the English language, but Portuguese and foreign languages are available at http://www.edudermatologie.com. It comprises 12 items, covering “worries” (6 items) and “beliefs” (6 items). Four response choices are offered, from totally disagree to totally agree, with points attributed to each one (0, 1, 2 or 3).^15^

European health literacy survey (HLS-EU) questionnaire is a validated tool for measuring the health literacy (HL) of the general population, also validated in the Portuguese language (HLS-EU-PT).^14^, ^17^ HLS-EU-PT includes 47 questions, which integrate three different health domains: healthcare, disease prevention, and health promotion.^14^, ^18^ For each item, patients rate the perceived difficulty of a given task on a four-category Likert scale (very easy, easy, difficult, and very difficult).^18^

A third questionnaire was applied to provide additional clinical data. This included demographic information such as gender, age, working in a healthcare setting, and disease characteristics such as duration of disease, family history of AD, personal history of asthma and/or allergic rhinitis, and previous AD treatments.

### Statistical analysis

TOPICOP© score was calculated as 100% times the sum of all responses divided by the maximum possible sum for the included questions, with a resulting score of 0 to 100% of TCS phobia.^15^ The score can be further categorized in low (≤ 23), intermediate (24‒50), and high (> 50).^19^ For HLS-EU-PT, a mean-based item score was calculated for each patient with a least 80% responses. The index score was transformed in a unified metric from 0 to 50 using the following formula: (mean–1)×50 ÷ 3. The final score is inserted in one of four levels of HL: “inadequate” (0‒25), “problematic” (> 25‒33), “sufficient” (> 33‒42), and “excellent” (> 42‒50).^18^ Results are expressed as percentages or means ± standard deviation, and statistical analyses were performed using SPSS version 22 (SPSS Inc., IBM-SPSS, Chicago, IL). Variables were tested for normality with the Kolmogorov-Smirnov test. For the univariate analysis, relationships between TOPICOP© score and covariate were evaluated using Pearson’s correlation test. When necessary, Student’s *t*-test was used. Pearson correlation and univariate linear regression tests were used to measure the effect of health literacy (provided by HLS-EU-PT score) on TCS phobia (measured by TOPICOP©).

Missing data were not replaced. Statistical significance was set at p < 0.05.

### Ethics

This study was approved by the Hospital Medical Ethical Research Committee. Measures were taken to maintain the privacy of the patients, by processing anonymously the questionnaires.

## Results

Patient characteristics are listed in Table 1. A total of 61 patients were included, 35 (57.4%) females, with a mean age of 20 ± 13.8 years. We had 25 patients (41.0%) with ≤ 15 years and 36 (59.0%) with > 15 years. Ten (16.4%) of the responders worked on a healthcare setting. The mean duration of AD was 12.5 ± 11.4 years. Other atopic diseases (asthma and/or allergic rhinitis) were present in 35 patients (57.4%) and 31 (50.8%) had a family history of AD. Most of the patients had mild disease (38 patients, 62.3%). Fifty-nine patients (96.7%) confirmed treatment with TCS. Other treatments used for AD are described in Table 1.

**Table 1:** Population characterization.

Population characterization	n (%)	Mean ± SD (min–max)
Age, years		20.0 ± 13.8 (1–49)
≤15 years	25 (41.0)	
>15 years	36 (59.0)	
Gender		
Female	35 (57.4)	
Male	26 (42.6)	
Duration of the disease, years		12.5 ± 11.4 (0–47.0)
Healthcare professional		
Yes	10 (16.4)	
No	51 (83.6)	
Family history of AD		
Yes	31 (50.8)	
No	30 (49.2)	
Personal history of asthma and/or allergic rhinitis		
Yes	35 (57.4)	
No	26 (42.6)	
Severity of AD		
Mild	38 (62.3)	
Moderate to severe	23 (37.7)	
Current and/or previous treatments		
Moisturizer	59 (96.7)	
Topical corticosteroid	59 (96.7)	
Topical calcineurin inhibitor	21 (34.4)	
Systemic corticosteroids	16 (26.2)	
Cyclosporine	14 (23.0)	
Methotrexate	6 (9.8)	
Phototherapy	5 (8.2)	
Dupilumab	2 (3.3)	
TOPICOP©		44.8 ± 20.0 (8.3–88.9)
Low (n, %)	9 (14.8)	
Moderate (n, %)	31 (50.8)	
High (n, %)	21 (34.4)	
Mean HLS-EU-PT		30.5 ± 8.5 (1.1–47.9)
Inadequate (n, %)	13 (21.3)	
Problematic (n, %)	26 (42.6)	
Sufficient (n, %)	16 (26.2)	
Excellent (n, %)	6 (9.8)	

TOPICOP© levels ranged from 8.3 to 88.9, with a mean score of 44.8 ± 20.0, which indicates a moderate level of TCS phobia. Nine patients (14.8%) had a low TOPICOP© score, 31 (50.8%) had an intermediate score and 21 (34.4%) had a high score.

HLS-EU-PT scores ranged from 1.1 to 47.9, with a mean score of 30.5 ± 8.5, considered a level of problematic HL. Inadequate HL was present in 13 (21.3%), problematic HL in 26 (42.6%), sufficient in 16 (26.2%) and excellent in 6 (9.8%).

No statistically significant difference was found for TOPICOP© score between the caregivers’ responders (patients with ≤ 15 years) and patients’ responders (> 15 years) (p = 0.189). Similarly, no statistically significant difference was found for TOPICOP© scores between healthcare professionals and other occupations, family history of AD, personal history of other atopic diseases, and disease severity (Table 2). TOPICOP© value was shown to be negatively correlated with HLS-EU-PT value (p = 0.002, *r* = -0.382,  *r^2^* = 0.146), with higher levels of TOPICOP© associated with lower HLS-EU-PT value (Table 3).

**Table 2:** TOPICOP© variation between groups.

Variables	TOPICOP (mean ± SD)	p-value
Age		
≤15 years	40.8 ± 20.8	0.189
>15 years	47.6 ± 19.1	
Healthcare professional		
Yes	34.5 ± 15.3	0.073
No	46.9 ± 20.2	
Family history of AD		
Yes	46.2 ± 22.4	0.583
No	43.4 ± 17.2	
Personal history of asthma and/or allergic rhinitis		
Yes	42.9 ± 20.8	0.379
No	47.5 ± 18.8	
Severity of the disease		
Mild	41.6 ± 19.2	0.099
Moderate to Severe	50.2 ± 20.3

**Table 3:** Pearson correlation of TOPICOP© with HLS-EU-PT.

Correlations	R	R^2^	p-value
TOPICOP© – HLS-EU-PT	-0.382	0.146	0.002

## Discussion

TCS is the first-line anti-inflammatory treatment for AD.^6^ Corticophobia may impair an adequate treatment regimen, leading to an uncontrolled disease with a negative impact on the quality of life. The right amount of TCS to use and frequency of applications are some of the concerns that should be addressed with these patients.^20^ The fear of TCS side effects may be a major concern in AD patients and their caregivers and is frequently implicated in TCS resistance.^9^, ^20^, ^21^ Cutaneous atrophy, purpura, telangiectasias, striae, perioral dermatitis, or acne are some of the adverse events that may occur with prolonged and inadequate administration of TCS.^22^ The increased percutaneous absorption may be responsible for systemic adverse effects, such as hypothalamic-pituitary-adrenal axis suppression and glaucoma, but the risk seems to be low.^23^, ^24^ In general, TCS long-term treatments are considered safe in both adults and children.^23^, ^25^, ^26^, ^27^

Nowadays, there is accessible access to the medical information in the media, such as newspapers, magazines, and the internet. However, much of this information is not produced by healthcare professionals and may erroneously induce the patient to undertreat with TCS. In fact, a Korean study showed that the internet (49.2%), television or other broadcasting media (45.2%), doctors/healthcare professionals (37.3%), and magazines/newspapers were sources of information associated with steroid phobia.^28^ Patients may have difficulties in selecting correct and trustworthy medical information.

TOPICOP© is currently the only validated score to assess TCS phobia. ^16^ In the present study, TOPICOP© questionnaire showed an average level of 44.8% in our population, which indicates an intermediate level of TCS phobia. Other groups reported similar levels of corticophobia levels.^16^, ^19^, ^29^ Stalder et al. reported a mean global TOPICOP© score of 44.7% in a prospective multicenter study conducted with 1796 participants from 17 countries.^16^ In a smaller study, Bos et al. found a TOPICOP© score of 44% in a group of 29 parents of patients with AD from the Netherlands.^29^ A French study by Dufresne et al. with 191 parents found a TOPICOP© score of 39.8%, slightly lower than the other studies, but these parents attended a therapeutic education program, which may have been responsible for the better results.^19^

In the present study, the authors’ main goal was to evaluate the association between TCS phobia and health literacy. The general HL index reported for Portugal was 33.0, and 11% of the Portuguese population had an “inadequate” HL score, and 38% had a “problematic” score.^30^ In the present study, we had a mean HLS-EU-PT of 30.5 ± 8.5 and 63.9% had lower levels (“inadequate” and “problematic”). Interestingly, we found a significant negative correlation between TOPICOP© score and HLS-EU-PT. Furthermore, lower HL was a predictor of higher corticophobia. This emphasizes the importance of HL in healthcare. The present results contrast with the findings of Dufresne et al., in which the literacy score was significantly associated with higher TCS phobia.^19^ Most of the corticophobic parents of their study had direct access to scientific and online resources and a higher TOPICOP© score was associated with completed years after graduation. However, these authors had a more homogenous group, including only parents of AD children (<18 years). The inclusion of a group of parents of younger patients and a group of older patients may be a limitation of the present study since it makes a more heterogenous cohort and may impact the general group analysis. Another limitation was the small number of patients in each group. Future studies with larger and more homogenous cohorts should be done in order to confirm the present findings.

Physicians should develop teaching techniques in order to properly educate their patients about the disease and the recommended treatment.^11^ However, TCS phobia is also present among healthcare professionals, and it may affect patients (or their caregivers) perspectives of TCS use and adherence.^29^, ^31^ Education of these professionals may improve patients' adherence to treatment. It was shown that having trust toward a physician is significantly associated with taking medication as directed.^9^ Education of pharmacy staff to provide patient counseling was also shown to be effective in reducing TCS phobia.^32^ Doctors should provide detailed written instructions on TCS regimens and answer any concerns that patients and caregivers may have.

The promotion of HL should be a priority in public health. The improvement of individual and social critical skills regarding their health will maximize the engagement of healthy behaviors.

## Conclusion

Corticophobia is an impediment towards an appropriate therapeutic regimen in cutaneous conditions such as AD. The easy accessibility to information on TCS and their side effects may paradoxically contribute to the fear and worries about them. Patients need to be capable of selecting and filtering reliable content. HL is responsible for the capacity and critical choice in health behaviors. The present study suggests that higher HL is associated with lower TCS phobia, but more studies are needed to clarify this association. To the authors’ knowledge, this study provides the first evaluation of TCS phobia in Portugal.

## Financial support

None declared.

## Authors’ contributions

Tiago Fernandes Gomes: Effective participation in research orientation; data collection, analysis, and interpretation; statistical analysis; critical literature review; preparation and writing of the manuscript.

Katarina Kieselova: Approval of the final version of the manuscript; data collection, analysis, and interpretation; manuscript critical review.

Victoria Guiote: Approval of the final version of the manuscript; data collection, analysis, and interpretation; effective participation in research orientation; manuscript critical review.

Martinha Henrique: Approval of the final version of the manuscript; data collection, analysis, and interpretation; manuscript critical review.

Felicidade Santiago: Study conception and planning; approval of the final version of the manuscript; data collection, analysis, and interpretation; statistical analysis; intellectual participation in propaedeutic and/or therapeutic management of studied cases; manuscript critical review.

## Conflict of interest

None declared.
